# The illusion of inclusion: contextual behavioral science and the Black community

**DOI:** 10.3389/fpsyg.2023.1217833

**Published:** 2023-10-30

**Authors:** Sonya C. Faber, Isha W. Metzger, Joseph La Torre, Carsten Fisher, Monnica T. Williams

**Affiliations:** ^1^School of Psychology, University of Ottawa, Ottawa, ON, Canada; ^2^Department of Psychology, Georgia State University, Atlanta, GA, United States; ^3^Behavioral Wellness Clinic, LLC, Tolland, CT, United States; ^4^Department of Cellular and Molecular Medicine, University of Ottawa, Ottawa, ON, Canada

**Keywords:** acceptance and commitment therapy, functional analytic psychotherapy, African Americans, Black Americans, racism, weaponization of policy, organizations, diversity

## Abstract

Anti-racism approaches require an honest examination of cause, impact, and committed action to change, despite discomfort and without experiential avoidance. While contextual behavioral science (CBS) and third wave cognitive-behavioral modalities demonstrate efficacy among samples composed of primarily White individuals, data regarding their efficacy with people of color, and Black Americans in particular, is lacking. It is important to consider the possible effects of racial stress and trauma on Black clients, and to tailor approaches and techniques grounded in CBS accordingly. We describe how CBS has not done enough to address the needs of Black American communities, using Acceptance and Commitment Therapy (ACT) and Functional Analytic Psychotherapy (FAP) as examples. We also provide examples at the level of research representation, organizational practices, and personal experiences to illuminate covert racist policy tools that maintain inequities. Towards eradicating existing racism in the field, we conclude with suggestions for researchers and leadership in professional psychological organizations.

## Highlights

- There is a mismatch between research on Black mental health and actual health needs.
- Black people need help for anxiety and PTSD more than substance abuse.
- Efficacy data on contextual behavioral therapies for Black people is lacking.
- Racist policy tools hinder inclusion and research on the Black community.
- Examples illustrate weaponization of policy and power hoarding in CBS communities.
- Conclusions provide practical steps for anti-racist organizational transformation.

## Introduction

“Power concedes nothing without a demand. It never did and it never will. … The limits of tyrants are prescribed by the endurance of those whom they oppress.”

— Frederick Douglass

### What is contextual behavioral science?

Contextual Behavioral Science (CBS) is a research paradigm that underlies the development of Acceptance and Commitment Therapy (ACT), Relational Frame Theory (RFT), Functional Analytic Psychotherapy (FAP), and other similar third wave cognitive-behavioral modalities that are grounded in Skinnerian behaviorism (Kohlenberg et al., 1993). CBS is a system of philosophical assumptions, scientific values, and methodological commitments that drive theory and practice. With its roots in Western scientism, CBS strives to be objective, empirical, and evidence-based (Vilardaga et al., 2009). In addition to being theoretical, CBS has been adopted by scholar-practitioners and other clinicians in the field of mental health and therefore has a direct effect on client care (Varra et al., 2008). There are several important facets of CBS, but, for the purpose of this paper, we will be using ACT and FAP as examples that are representative of the issues at hand.

### Acceptance and commitment therapy

ACT is an approach that is based on Buddhist principles and values such as mindfulness, cognitive defusion, and coping with distressing thoughts and uncomfortable feelings. It encourages people to embrace their current thoughts and feelings rather than avoid them or feel guilty for them, which in turn helps to resolve symptoms associated with a range of mental health conditions such as anxiety, depression, OCD, addiction, and substance abuse, which have all been found to benefit from ACT (Wetherell et al., 2011; Bramwell and Richardson, 2018; Osaji et al., 2020). In addition to targeting thoughts and cognitive processes, ACT also combines strategies and techniques grounded in behavioral therapy (e.g., meditation, mindfulness) through emphasizing the self-acceptance to develop psychological flexibility, which is one’s ability to cope with change and try new things (Fledderus et al., 2013).

The other major tenet of ACT in addition to acceptance of thoughts and feelings is commitment. ACT encourages clients to become committed to acceptance as well as certain behavioral techniques and, through this model, directs clients to act in ways that allow them to face problems directly rather than avoiding stress (Hayes et al., 2013). This can look like committing to actions that help facilitate experiential learning and embracing challenges with a goal being to exercise psychological flexibility. The opposite of psychological flexibility is called experiential avoidance (EA), which is characterized by adversity to change and resistance to trying new things (Biglan et al., 2008). This is when people avoid unpleasant thoughts and feelings, which is believed to help perpetuate symptoms associated with psychopathologies such as anxiety disorders, OCD, and PTSD.

It is important to note, however, that ACT has not been sufficiently studied in all populations and data supporting its efficacy among diverse groups is lacking. While ACT may help a variety of specific behavioral problems, it does not address the fact that individuals from different ethnoracial backgrounds may experience the approach differently (Sobczak and West, 2013). Thus, there is a need for clinicians to be ethnoracially-sensitive when it comes to using ACT with diverse clients.

### Functional analytic psychotherapy

FAP is a behaviorally based, experiential and relational approach to psychotherapy in which therapists focus on dyadic interactions in session to shape the interpersonal behaviors, develop emotional awareness, and practice the self-expression necessary for clients to create and maintain close relationships with others (Kohlenberg and Tsai, 2007). In FAP, clinicians model practicing vulnerability and honesty with clients with the goal of identifying a number of different clinically relevant behaviors, which are targets for change (CRB1), behaviors that demonstrate clinical improvements (CRB2), and client interpretations of behaviors (CRB3). Given that interpersonal challenges are common problems across a range of disorders, FAP has broad applicability (Wetterneck and Hart, 2012).

FAP is similar to many other CBT interventions because it focuses on making measurable behavioral change and includes assignments for clients to complete between sessions, but the distinguishing characteristic of this treatment is its reliance on building a strong therapeutic relationship as the primary vehicle for client change. In FAP, a genuine and corrective therapeutic relationship serves as the basis for clients to learn healthy communication and relating, and to repair dysfunctional patterns of interpersonal functioning they may have outside of therapy. This dyadic relationship is collaborative with respect to treatment plans and powerful in promoting learning and change, fostering motivation, and keeping clients engaged in treatment (Miller et al., 2015).

### Purpose of this paper

The authors of this paper are a diverse group of clinicians and researchers, living and working in varied cultural contexts. The first author lives in Germany and is an experienced neuroscientist and pharmaceutical professional, specializing in clinical development and social justice issues. The second author is a first generation African American, researcher and licensed clinical psychologist who teaches and provides clinical supervision at Georgia State University, a top research university in the Southeast United States. The third author is a queer White person completing their doctorate in experimental psychology at the University of Ottawa, with a Masters from Harvard University in Religious Studies. The fourth author is an African American behavioral specialist and psychedelic integration therapist in training who completed his Master’s in Behavioral Psychology at Pepperdine University. The senior author is a Black clinical psychologist, former member of the Association of Contextual Behavioral Science (ACBS) and Canada Research Chair for Mental Health Disparities at the University of Ottawa, where she studies disparities and racialization.

Collectively, the authors have been concerned for many years about the apparent lack of CBS scholarship focused on Black people and about the lack of representation of Black people in ACBS, notably in the ACT and FAP communities. The purpose of this paper is to discuss how those who have developed these modalities have not included the voices and needs of Black people in their work and in the leading professional organization that supports CBS.

The lead authors of this paper are Black women who count themselves fortunate to follow the steps of their foremothers in speaking out for social change. Notably, as soon as Black American women had access to higher education in the late 19th century, they also began working from within educational institutions as a force for justice (Bell et al., 2021). We note that it is difficult to get papers about the impact of race on power published due to racial bias in the publication and peer-review process (Buchanan et al., 2021; Strauss et al., 2023). The same forces that minimize and exclude Black people in education, psychology, and professional organizations also attempt to silence Black people when they speak up against mistreatment and will assert that Black people are unqualified to provide a true account of their own experiences. We reject this notion and recognize it as yet another form of anti-Black oppression.

The structure of this paper is first to present the empirical evidence regarding the inclusion of Black Americans in research, research priorities, and as participants in studies using these methodologies. Second, we show with examples the power dynamics of the ACBS structure through reports about treatment of Black people, review indications of a lack of inclusion, and note initial inroads for inclusion. Finally, we illuminate racist policy tools used by ACBS through individual and personal stories of Black professionals about their experience in this community, and we offer suggestions for beginning the healing process and promoting positive change in representation and inclusion in the field. This paper is not meant to be a systemic review, rather a critical assessment of Black mental health needs and experiences within the context of CBS.

### Race and racism

Racism is a system of beliefs, practices, and policies based on race that operate to provide advantages to those with historical power – White people in the US and most other Western nations (e.g., Canada, Western Europe, Australia, etc.). Race is a made-up social construct used to group people based on shared physical features and presumed ancestry. Race has no biological basis and was born from White supremacy, an ideology that presumes the superiority of White people and inferiority of People of Color (Smedley and Smedley, 2005; Haeny et al., 2021).

Racism, with its roots in White supremacy, operates hierarchically, with White people on the top, Black people at the bottom, and Asian people generally falling in between. This hierarchy is mediated by skin color (colorism), whereby people with darker skin of any race are considered lesser than lighter skinned persons of that same race. Colorism causes people to be devalued in the US, Canada, Europe, Latin America, and many Asian countries (Dixon and Telles, 2017; Jablonski, 2021). For example, White-skinned people in Latin American countries have privilege over darker-skinned persons, even though in the US we tend to consider them all Hispanic. Even so, in the US, White Hispanic Americans are still advantaged over their darker-skinned counterparts, with disparate outcomes (Cuevas et al., 2016). Hispanic used to be considered a racial group in the US, but it is now considered an ethnic group instead, as per the US Census Bureau, illustrating how race is determined by social decisions and not biology (Borrell and Echeverria, 2022).

Anti-blackness is a type of racism focused specifically against Black people. It has been described as a theoretical framework that illustrates and explains the dehumanization of Black people, including disdain, disrespect and disgust of all things connected to Black people (Bell et al., 2021). The field of psychology perpetrates the same anti-Black biases, stereotypes, and hatred that exist in the rest of our society. Due to experiences with anti-Black racism, Black people in Western society have a well-founded fear of discrimination, a mistrust in health service systems, and suffer due to inaccurate myths about Black people (e.g., pathological stereotypes). Most medical school curricula frame race as a “biological risk factor” rather than a social one, which implies that disparities in health are inborn and the differences we see in mental health are due to natural causes and can be explained without implicating racism (Haeny et al., 2021). This misconception harms both the treating clinician and the client of color because it pathologizes race rather than racism, whereas it is the racism that is the risk factor (Noonan et al., 2016; Alang, 2019). Only addressing the stigma of a mental health disorder without addressing the racism does a disservice to the person seeking treatment (Alang, 2019).

Deficits in empathy have been identified as a correlate of racist behavior, and multiple studies including both White and Black samples, document that people exhibit greater empathic resonance to those with a similar skin color (Azevedo et al., 2013; Hoffman et al., 2016; Harjunen et al., 2021). The converse is true regarding White people’s perception of (and perhaps, ability to empathize with) the pain experienced by Black people. Specifically, brain imaging reveals anti-Black racial biases wherein the pain of Black individuals is perceived to be less distressing and more tolerable than the pain of White people or even a purple “space alien” (Berlingeri et al., 2016; Harjunen et al., 2021). This racial bias in empathy has been associated with racial bias in social behavior as well (Han, 2018). In order to work effectively across racial differences and even find motivation to conduct research that will benefit people from different racial groups, cross-racial empathy is vital. These racial biases are with and in us due to our learning history and must be overcome to become ethically and culturally competent researchers and therapists.

In addition to individual biases, organizations and institutions also carry racial biases, and these are built into the rules, policies and procedures. These constructs, called institutional racism, function in the background to arrive at discriminatory outcomes without a single individual needing to engage in explicitly biased behavior. This has been well documented in education, academic publishing, and the discipline of psychology (Williams, 2019; Avery et al., 2022; Dupree and Kraus, 2022).

For the purposes of this paper, we use the term Black in reference to African Americans, dark-skinned Africans, and all people who are descendants of the African diaspora (of partial or full African ancestry). White is being used to describe people who trace their origins to Europe, have lighter skin, and in general who do not have any visible Black African, Asian, or other Indigenous ancestry (Haeny et al., 2021).

## Mental health priorities in the Black community

### Needs of the Black community

A survey conducted by the Kaiser Family Foundation and ESPN’s “The Undefeated” explored African Americans’ experiences of being Black in America, utilizing a dual-frame (landline and cell phone) random digit dial methodology (N = 777). It was found that a majority of Black men and women, regardless of age, income, and education, believe it is a bad time to be Black in America, with increases in this percentage being 37% among Black men and 44% among Black women from 2006 to 2020 (Hamel et al., 2020).

Authors of this survey suggest that the disproportionate impact of the COVID-19 pandemic on Black families and the frequent media coverage of police violence towards Black Americans in the summer of 2020 may impact the perception of personal belonging in America. When exploring personal and familial concerns amongst Black respondents, more than a third stated financial concerns and COVID-19-related concerns as their top priority (Hamel et al., 2020). Authors found that in addition to housing affordability, lower cost of healthcare, higher paying jobs, and college affordability, two-thirds of respondents prioritized racism as a major concern (Pougiales and Fulton, 2019).

These stressors can lead to mental health disorders (e.g., racial trauma) that disproportionately impact the Black population in the US (Williams et al., 2021b). Therefore, the mental health priorities of Black communities cannot be expected to be identical with those of White communities.

### Differences in mental health between Black and White communities

It is well-documented that there are racial differences in mental disorders in the United States between Black and White populations. Black Americans on average experience higher rates of psychosocial stressors than White Americans, but at the same time historically had the same or better overall mental health than White individuals (Louie and Wheaton, 2018; Thomas Tobin et al., 2022). The validity of this paradox has been consistently demonstrated in adult populations, however, not only do Black communities exhibit lower levels of mental health disorders, but there are also racial differences in the prevalence among categories of mental health diagnoses between Black and White Americans. These racial differences also differ across generational cohorts.

It is important to keep in mind that, although Black people have similar or lower rates of common mental disorders than White individuals, according to the existing published studies, when they do suffer from mental disorders these are of a greater duration, more severe, and more disabling among the Black population; and in addition, Black Americans are less inclined to find and receive competent mental health services. This means that the unmet mental health care needs of Black Americans exceed that of the White community (Noonan et al., 2016; Alang, 2019).

**Figure 1:**
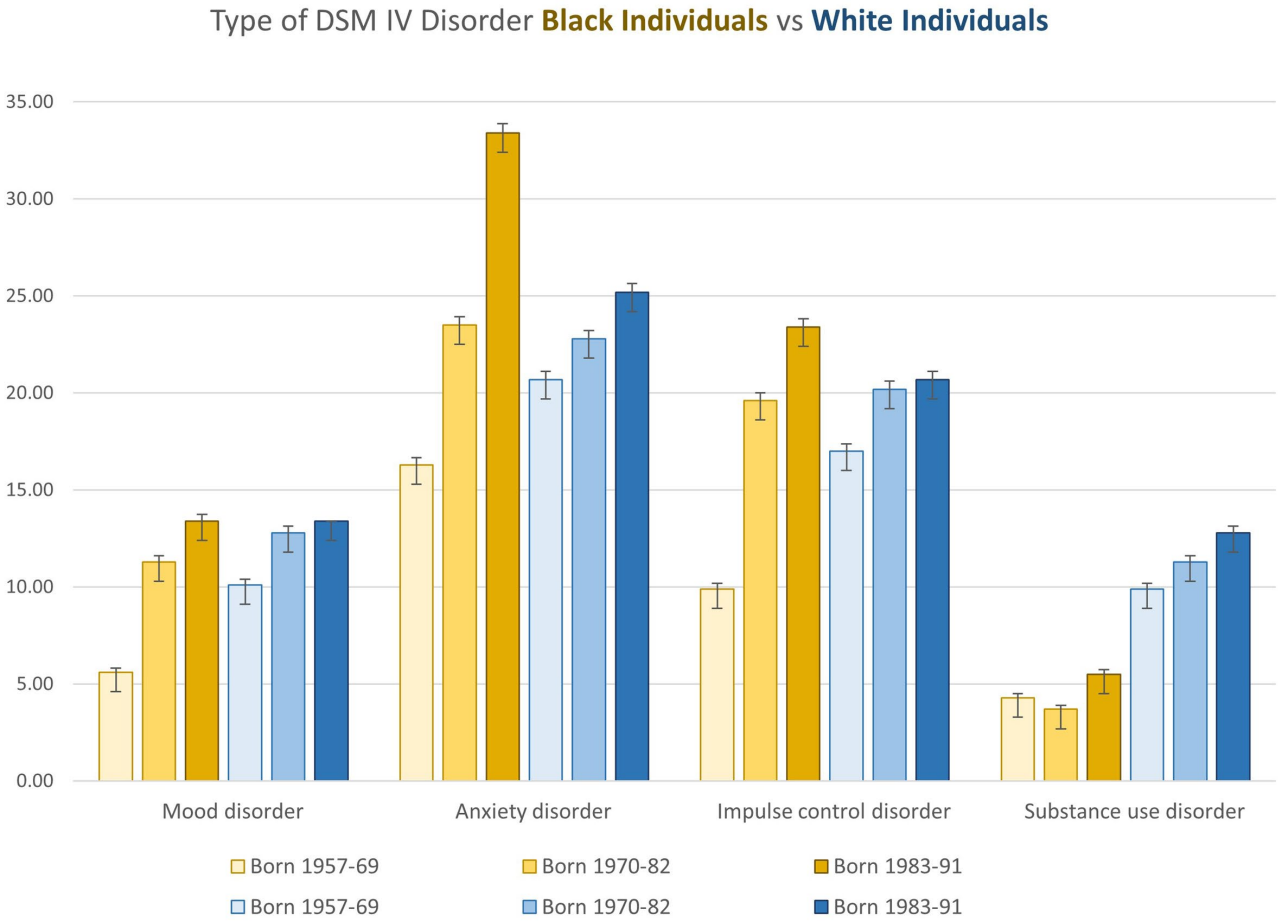
Characteristics (Percentage and Standard Deviation of Adolescent Survey Respondents in a Study of Race, Birth Cohort, and DSM-IV Mental Disorders, by Race and Cohort, National Comorbidity Survey Adolescent Supplement (2001-2004) and National Comorbidity Survey Replication (2001-2003). Mood disorders: major depressive episode, dysthymia, bipolar disorder I, or bipolar disorder II, Anxiety disorders: panic disorder, agoraphobia, social phobia, general anxiety disorder, post traumatic stress disorder, or separation anxiety disorder, Impulse control disorders: oppositional defiant disorder, conduct disorder, attention deficit disorder or intermittent explosive disorder, and Substance use disorders: alcohol abuse, alcohol dependence, drug abuse or drug dependence. Plotted from a table in Louie and Wheaton (2018).

Younger cohorts of Black people have higher odds of being diagnosed with anxiety than White people, and the magnitude of anxiety disorder is higher in younger cohorts (Louie and Wheaton, 2018). In addition, for younger generations, the rate of increase in anxiety disorders exceeded the rate for White individuals over the same time period (Figure 1). The sharp increases in the numbers of Black Americans suffering from anxiety disorders is attenuated for mood and impulse control disorders but also significant.

In comparison, Black Americans do not have substance abuse disorders in either past or current generations to the same extent as White Americans, although Black Americans are punished disproportionately more severely by society for drug use (Alexander, 2010). A recent study of 37,860 patients in fact found that Black mothers had a higher likelihood of receiving a drug test during delivery compared with White patients, regardless of their substance use history. Their chances however of a positive test result was *lower* than for White mothers and other racial groups, demonstrating the greater societal emphasis on unearthing and addressing substance use in Black communities based on racist stereotypes (Bor et al., 2018; Jarlenski et al., 2023). A simple PubMed search points to a mismatch between studies on Black mental health and the actual mental health needs (Black “substance abuse” yields 2027 studies versus Black “anxiety disorder” resulting in 297). There is an overrepresentation of CBT RCTs on substance use as compared with all other mental health issues when looking at trials specifically designed for African American participation. This is indicative of a mental health establishment with its own implicit biases in regard to Black people (Jarlenski et al., 2023). Mental health provision may disturbingly be set up to offer services in instances where society at large perceives Black individuals to need treatment (substance abuse) rather than in areas where they are more likely to require competent mental health services, such as anxiety and mood disorders (Smith et al., 2022). It is well-documented that, due to a lack of empathy, White clinicians often misinterpret mood symptoms, resulting in over diagnosis of Black people with disorders such as schizophrenia compared to White patients (Schwartz and Blankenship, 2014; Smith et al., 2022; Faber et al., 2023a).

The increases in diagnoses of impulse control disorder and anxiety in particular for Black cohorts, can be explained in part by historical changes that occurred between 1957 and 2004 (Louie and Wheaton, 2018). Rates of child poverty increased between 1974 and 1983 for both Black and White Americans but had a more profound effect on Black individuals, at the same time the unemployment rate for Black communities grew from 1962 to 1985 from 13 to 22%, with over 38,000 Black school workers fired in retaliation in the aftermath of school desegregation (Lutz, 2017). The percentage of Black parents furthermore with a college degree declined from the middle cohort (22%) to the youngest cohort (19%), and between 1970 and 1990 the number of single mother households increased among Black women with the Black-White difference in nonmarital births growing from 32 to 50% in this period. Many of these trends are a result of the US war on Black people which resulted in imprisonment for Black men in America increasing three-fold between 1969 and 1999. This devastated families and communities who were left to suffer the socioeconomic and emotional consequences of targeted mass incarceration (Alexander, 2010; Louie and Wheaton, 2018).

Examining the subcategory of posttraumatic stress disorder (PTSD), which was classified as an anxiety disorder in the NCS-R, there are also differences between Black and White populations. Three studies examining the prevalence rates among the US population of PTSD using large national samples weighted to the population reported similar results (Roberts et al., 2011; Alegría et al., 2013; McLaughlin et al., 2019). In contrast to depression, anxiety, and substance disorders which are lower among Black Americans, PTSD has a higher prevalence among Black people, with a higher odds of lifetime PTSD than White Americans (OR = 1.25) likely due to greater exposure to adversity and discrimination across the lifespan relative to White individuals (Andrews et al., 2015; McLaughlin et al., 2019). Ongoing disparities in treatment indicate a need for investment in culturally competent treatment options (Roberts et al., 2011; Williams et al., 2022).

PTSD is defined by the DSM-5 as a process in which a person has an initial exposure (directly or indirectly) to trauma, followed by symptoms rooted in the exposure causing multiple disruptions in the daily life of the one suffering from the disorder. The DSM-5 guidelines were updated to be more inclusive of the harmful effects of newer aversive or chronic forms of bias than were the previous fourth edition (i.e., chronic exposure to aversive stimuli). This new expanded definition for PTSD provides some room for the reality that the Black community already lives with higher rates of PTSD, in part from chronic stressors in the media (Himle et al., 2009; Smith et al., 2022). A more significant emotional injury for Black people is racial trauma, which is related to PTSD but often has a different etiology and treatment requirements (Williams et al., 2021a; Smith et al., 2022). Racial trauma has been studied for over 20 years by Black scholars, but there has been scarce funding for empirical studies, and only recently have established clinical tools and diagnostic criteria been published (Carter, 2007; Williams et al., 2018).

Faced with these differences, it stands to reason that mental health priorities for Black people should be culturally relevant and cater to those disorders with high prevalence in the population and those that are rapidly increasing. One of the major issues in examining data from studies with data that were collected in the 2000’s is that, at that time, there was no recognition or validated measure to assess the effects of racial trauma. The rise in “anxiety disorders” documented in publications is likely to hide the existence of racial trauma which was not considered at the time of these studies.

The appreciation of racial trauma requires a therapeutic remedy whose parameters are only now being defined (Metzger et al., 2021; Williams et al., 2021a, 2022). The relevance of CBS for Black people in the future must depend on how thoroughly Black-specific therapeutic structures (paradigms) and skills are imparted to those therapists who will be treating these patients. In effect, all CBS therapists must update their knowledge and vocabulary and seek the necessary retraining and continuing education on these new protocols to be considered proficient to offer meaningful therapy to POC suffering due to racialization.

### Why younger cohorts experience greater anxiety

Studies using more recent data from a 2011–2015 National Survey surveyed Black adults with unmet mental health needs (N = 1,237) and highlight some reasons for greater anxiety among younger cohorts. One such study surprisingly found greater unmet needs among Black cohorts with higher education levels (Alang, 2019). Specifically, Black college students ages 18–25 reported stigma as a significant barrier to professional mental health services. Furthermore, across all ages, employment and college education were associated with increased odds of experiencing stigma wherein the more education a Black person had, the greater the increase in the reports of marginalization and ineffective treatment (Alang, 2019).

Younger cohorts have higher education levels and their exposure to White institutes and power structures are currently greater than in previous generations. The fact that younger cohorts are coming into competition with White cohorts for middle class jobs (which in older generations were cordoned off for the White male population) means that Black exposure to professional malice, envy, rancor, resentment, and disaffection is higher than in previous generations (McDonald et al., 2018). Essentially, because Black individuals in the middle class compete, work and are evaluated alongside White persons, and exposure to racial discrimination increases with upward class mobility, the issue of double discrimination based on race and social status exacerbates the resulting mental health consequences of racism (Noonan et al., 2016; Alang, 2019).

Taking this information into account, it may be easier to understand why a Black person may think that a White mental health provider may not have their best interests in mind or even be implicitly working against the client’s true mental health needs (Bergkamp et al., 2022).

### Meeting the needs of the Black community: how can CBS move these goals forward?

CBS is a research paradigm underpinning therapies ultimately designed to improve treatment of mental disorders. The first study on ACT was published 30 years ago in 1986 and since then, as of 2019, over 325 randomized controlled trials have been carried out using ACT. More than 20 meta-analyses have been published as well, with most studies reporting results that favor ACT with no contraindications for use (Gloster et al., 2020). Although ACT and similar CBS intervention strategies such as FAP have been shown to be effective for treating and managing symptoms associated with a number of mental health conditions, we need to determine if there is evidence for its efficacy and safety in diverse populations, and in particular, racially marginalized individuals. It is possible that ACT and FAP could be helpful to members of Black communities, yet treatment protocols may still need to be tailored and customized for work with clients in a way that creates an ethos of ethnoracially and culturally-safe care.

## Relevance of ACT for the Black community

Although ACT has been used in different countries, it cannot be assumed that outcomes are generalizable between US populations of non-White versus White persons given the mental health disparities already documented between US Black and White communities. Black Americans face the same psychological stressors as every other racial group in addition to daily racial discrimination and microaggressions, thus the efficacy of ACT must be tested outside of mostly White frameworks.

### ACT RCTs for Black mental health

It is important to distinguish between the few ACT randomized trials that include people of color and the even smaller subset that focused specifically on their mental health. In a recent study, it was found that papers focused on the mental health of non-White individuals were underrepresented, and those specifically for African Americans dropped to only a handful. Out of 100 assessed ACT studies published between 2002 and 2022 in 34 different journals (25 were excluded due to missing demographic information), the remaining 75 included 10,914 participants. Among these participants, 8,010 (73%) were White and 1,212 (11%) were Black. Most studies (84%) focused on ACT interventions for specific clinical concerns, including anxiety, general distress, reducing stigma, and working with mental health professionals. Ten (13%) studies had majority non-White samples and one study had a fully Japanese sample. The diversity of targets in the selected studies made it difficult to compare results based on race. While the studies reviewed included Black individuals, the relatively small sample sizes of this population across studies precluded the ability to infer the effectiveness of ACT interventions for this group without subgroup analyses. Most studies were not designed to assess mental health in Black communities. Notably, none of the studies provided analyses of outcomes based on ethnoracial differences and only eight (Table 1) studies (11%) had a majority of participants of color or reported on differential outcomes for racial and ethnic minorities, with two of these studies authored by individuals from underrepresented racial and ethnic groups (Misra et al., 2023).

**Table 1:** ACT studies featuring participants of color.

Ethnicity or race included	Eight ACT randomized controlled trials including identifiable Non-White samples	Reference
Majority Non-White	Non-White participants in a study comparing ABBT with CT for test anxiety demonstrated improved test scores after treatment.	Brown et al. (2011)
All Japanese ethnicity	Japanese college students studying in the United States experienced improved mental health and increased psychological flexibility after 2 months of ACT bibliotherapy	Muto et al. (2011)
50% Black Mental Health Professionals	Combining ACT with applied behavioral analysis training resulted in improved general distress, particularly in those who actively practiced the ACT skills, and this improvement was greater than applied behavioral analysis alone, especially for those who were initially more distressed	Bethay et al. (2013)
Over 50% women of color	The effectiveness of cognitive-and acceptance-based coping skills to avoid consumption of sweets was compared, and results showed that acceptance-based consumption group experienced reduced rates of cravings and level of consumption, especially for those that reported greater awareness of the food environment and increased emotional eating	Forman et al. (2013)
67% Black college age men	In men with gambling disorder, increased psychological flexibility and present-moment awareness was experienced after receiving 8 h of individual sessions of ACT, compared with no treatment	Dixon et al. (2016)
78% POC 22% White participants	A study comparing ABBT with treatment as usual found that ABBT improved attendance to medical appointments, illness-related experiential avoidance, willingness to disclose HIV status, and number of HIV disclosures in people living with HIV	Moitra et al. (2017)
51% POC 49% White	Both ACT and CBT were found to improve symptoms in a study comparing them for social anxiety disorder, although this improvement was more prevalent in the CBT group	Herbert et al. (2018)
27% Black 25% Latine	A majority non-White sample of individuals suffering from mild to moderate traumatic brain injury had improved psychological distress, increased psychological flexibility, and committed action after receiving eight sessions of ACT	Sanders et al. (2022)

Although inclusion of Black participants as per the US Census is perhaps adequate, the participation of marginalized ethnic groups per study is too low to assess racial differences in treatment response, and the studies were not designed to do so. This makes the generalizability of the pooled data questionable (Printz et al., 2019; Misra et al., 2023). Of these studies, two specifically examined racial differences and found no differences in treatment efficacy.

A more recent search found 64 studies in which Black patients again were included, however only a handful had a specific focus on mental health of the Black community in the US. Of the few, one is an anti-smoking cell phone app, with findings derived from a secondary analysis. As Black Americans generally suffer from substance abuse at lower rates than White Americans, the more salient mental health need for Black Americans is therapy for anxiety, rather than substance use, yet there is an overrepresentation of the former compared with the latter type of research (Louie and Wheaton, 2018; Banks et al., 2021; Jahn et al., 2021; Santiago-Torres et al., 2021).

A webpage of ACBS identified another study conducted on a non-White population: Lundgren et al. (2006) conducted an RCT showing that a 9-h ACT protocol reduced seizures in people with epilepsy, while a placebo had no effect. The participants were all non-White South Africans from majority ethnic groups with low socio-economic status living in a residential center. Although ACBS inclusion of this on their “minorities” page implies it may be relevant, since these South Africans are not minorities and do not categorize themselves according to American racial norms, the actual relevance of this study for what the title of the webpage terms “minorities” is unclear.

### Use of ACT for Black people in other studies (not RCTs)

We conducted a search of the APA PsycInfo Database for additional psychology papers using ACT with Black Americans and found only a handful of relevant journal articles and one book chapter. The book chapter is about utilization of ACT in a case study of a single first-generation STEM African American woman with academic, financial, emotional, and familial stressors (Gant, 2020). The case represents an example of how African American college students’ access to STEM careers remains low and highlights how the psychological flexibility model in ACT can address the unique challenges of Black clients if clinicians focus on holistic growth in the counseling process.

The first ACT peer-reviewed paper examines the feasibility of using ACT in 9 African American adolescents with ADHD, learning disorders, or behavior problems to improve congruence between behaviors and values (Murrell Steinberg et al., 2014). The study (although likely not adequately powered due to its small sample size) found significant reliable change on the Behavior Assessment Scale for Children, suggesting that ACT could be effective in improving behavior and may have clinical use in youth.

The second paper included 11 adolescent patients with depression, only 5 of whom were African American (4 identified as multiracial, and 2 were White). All were given 3 sessions of motivational interviewing and 12 sessions of ACT, and while also underpowered, the results showed some improvements in depressive symptoms (Petts et al., 2017).

The last paper identified in our search was a recent pilot study (N = 20) documenting pre-post decreases in internalized racial oppression and shame, and psychological distress in Black women treated with ACT (Banks et al., 2021). ACT is considered a method which is particularly effective for anxiety disorders, and as such this paper is much needed and timely.

In examining these few papers, we see that although the samples were primarily African American, most were not *a priori* designed for African Americans. Further, the sample sizes were very small and the data presented was preliminary. Although these studies make useful contributions, this data is not sufficient to establish that ACT is a relevant and useful technique that will improve the mental health of African Americans, especially as some of these treatments address stereotypical issues (school difficulties, behavior problems) as opposed to priority areas as identified by research or Black communities. The lack of Black participation and leadership in the CBS community contributes to the lack of research relevant for these populations.

### ACT to reduce racial prejudice

There is some limited research that ACT can help reduce racial prejudice, which is an issue of great concern to Black Americans. In Lillis and Hayes (2007), undergraduates (*N* = 32) enrolled in two separate classes on racial differences were exposed to ACT exercises and an educational lecture from a textbook on the psychology of racial differences in a counterbalanced order. In this study, only the ACT intervention was effective in increasing positive behavioral intentions after 1-week follow-up. These changes were associated with other self-reported changes that fit with the ACT model, such as greater acceptance and flexibility.

Using some of these same techniques, Williams et al. (2020a) developed the Racial Harmony Workshop (RHW), to reduce racial biases and microaggressions and promote interracial connection among college students in a pilot study. The RHW was designed to increase connectedness across racial groups, using principles and techniques from ACT and FAP. Results indicated positive benefits for both Black and White participants (N = 44), including improved mood and increased positive feelings towards Black people for the White students, as well as increased ethnic identity for the Black students. White students in both conditions showed a decreased likelihood of committing microaggressions, and those in the RHW condition also showed a decreased likelihood of having microaggressive thoughts and increased gains over time. A related pilot study to reduce racism in medical students found similar results (Kanter et al., 2020).

It is encouraging that research is being carried out to use ACT creatively to reduce the effects of racial prejudice and bias in society. This is an area that has a pressing need in today’s environment that remains understudied, but more problematic is that there are no reports of it actually being implemented in organizational structures (juries, police forces, schools). Further, the fact that there are only 3 papers and no grants for scale-up studies since 2007 speaks to lost opportunities and lack of will to follow-through on these promising initial reports (Gordon, 2022).

## Relevance of FAP for Black people

Given the increasing numbers of Black people requiring and seeking mental health care, there is a growing need to enhance cultural competence in therapeutic interventions. Because of its emphasis on reciprocal vulnerability and empathetic responding, FAP is an excellent modality for incorporating sensitive cultural factors into ethnically and racially diverse client-therapist dyads (e.g., Vandenberghe, 2017; Williams et al., 2020b). Use of FAP can make treatment more genuine and relevant for underserved and racialized clients. An assessment of functional and non-functional behaviors of both therapists and clients can be examined from a FAP perspective and used to build alliances across differences, explore experiences of racism and discrimination, identify biases in the therapist or clients, and resolve microaggressions in sessions which can otherwise rupture the therapeutic alliance (Miller et al., 2015). Nonetheless, there has been a notable lack of FAP publications focused on the Black experience. In light of the unique mental health history and specific needs of the Black community as outlined above, it is worth examining how FAP has been failing the Black community and analyzing the context of these experiences through the lens of its Black members.

### Retaliation at FAP/ACT workshop and policy weaponization

Although the hope is that mental health clinicians will be more sensitive and aware than most, even professionals who aspire to be anti-racist can cause harm if their biases are unchecked. This can only be remediated through purposeful anti-racist work, otherwise these prejudices cause harm even within the CBS community. It is often difficult for those who do not experience anti-Black racism to envision how these processes actually play out, therefore we provide real examples to illustrate how these issues affect real people and to document these transgressions in the context of the historical struggle for Black equality. In the first of three examples (Box 1), all of which permission to share has been granted by the victim, an anti-racism training was organized by diverse trainers, and the 5 expert clinicians gathered on the West Coast to lead a three-day workshop that included several graduate student clinician attendees and therapists of color.

BOX 1 **Perspective: an incident of racism from a Black educator.**

Disturbing issues with a White FAP-certified trainer started in the months prior to the event, when this individual demanded and was eventually allowed to take charge of a FAP/ACT diversity workshop, initially conceived and organized by a Black trainer.

While sharing his own personal antiracist journey during the event, he refused to acknowledge the role of White privilege in his success and described himself as “a crusader.” When some participants of color objected to this, he became defensive. At the end of the day, he was confronted by other participants at the event and the White trainer became aggressive, such that the other four trainers had to intervene on the first evening of the workshop. The observed behavior was experienced by participants as rude, insulting, and argumentative. Several of the participants were deeply hurt and a few students cried. The problem was so severe that he was removed as a trainer from the workshop for the second day – a unanimous decision by the other four workshop leaders. Although the White lead trainer was brought back in a lesser capacity for the final day, he persisted in his aggression by making unprovoked bizarre and spiteful comments toward the Black trainer.

This particular individual was now known to the CBS community to be unsafe, but he persisted in trying to conduct more of these trainings. Although academically qualified, the trainer was not consistently capable of cultural humility. This individual shortly thereafter applied to conduct the same ACT/FAP anti-racism workshop for an ACBS conference, without involving any trainers of color. Because of the harm previously caused by this individual, the Black trainer tried unsuccessfully to persuade the White trainer not to proceed, voicing fears that more harm would come to vulnerable participants.

Apart from the Box, both Black and White involved FAP persons were consulted on the wording of the incidents.

Here it is worth pointing out using this illustrative example (Box 1) how the organization within the current system functioned to protect the White individual who transgressed at the expense of harm to the Black trainer and participants of color (i.e., Gaertner and Dovidio, 2008; Okun et al., 2019). In an organizational shortcoming, a proposed ACT/FAP follow-up workshop by the White trainer (Box 1), who had publicly demonstrated a lack of cultural-sensitivity, was *accepted by ACBS without POC involvement*. After being confronted by another trainer due to the previous issues, this person refused to step down from the leadership of the workshop and did not add any trainers of color to the planned training.

Following the failure of professional mediation, several of the trainers and participants who had been impacted by the lead trainer’s aggressive behavior at the previous workshop reported their concerns directly to ACBS conference organizers, who expressed shock upon realizing their oversight. They also said, “If we had known it was all White people teaching, we would not have approved it,” and after some extended deliberation cancelled the workshop for that summer. ACBS had invited the Black trainer (Box 1) to lead the workshop since many people had already registered, but the Black trainer declined, fearing retaliation from the ousted White trainer. As an alternative, they discussed inviting the Black trainer for the subsequent conference. When the next ACBS was planned, however, the Black trainer was not invited to host a workshop but instead told that they were considered *inadequately qualified* by the conference organizers, despite national prominence and having written a CBS book on this topic. This kind of retaliatory action is often observed when whistleblowers shine light on organization misdeeds (Ahern, 2018). While the Black trainer missed out on this opportunity, from a systemic level, it is important to note that the White trainer continued as a CBS trainer and retained his title as an ACBS Fellow and Certified FAP Trainer, despite a subsequent complaint from another co-trainer of color who was also worried about him causing harm to people of color.

This example is important because it illustrates how policy and power dynamics in organizations are used to covertly discriminate (Okun et al., 2019). Most prominent here is “*weaponization of policy*” which can take two forms. In the first, an organization has a lack of clear standards or qualifications for a position, as seen above. This is used to plausibly deny the position to a minoritized person and permits non-White experts to be held to a higher standard than White insiders. If the policies are unclear or there are no written policies, those entrusted with enforcing the rules have the power to apply different standards to arrive at unjust outcomes (Okun et al., 2019; Faber et al., 2023b). The flip side of this is having a defined standard that is only enforced for minoritized individuals, these can be written or unwritten rules. An example of an unwritten rule that applies only when writing about issues such as Whiteness and racism and affects primarily people of color, is the higher burden of proof, which increases the requested references by reviewers, likelihood of rejection, and time to publication (Avery et al., 2022).

The other tool observed here is *power hoarding*, which includes the withholding of information such that decision-making is clear to those with power and unclear to those without it. Finally, these are *aversive racism* tactics. A White supremacist culture organization will use these policies repeatedly to arrive at a plausibly deniable discriminatory outcome without explicitly targeting any one individual, although often harming and putting non-White people at a disadvantage (Okun et al., 2019; Chen et al., 2021). Aversive rules are based on misdirection or deception and can be difficult to change or perceive because they are constructed to appear, as if they were just, although their outcomes are discriminatory. It is like saying that everyone can appeal to their legal advisor, diversity office, or university, but if only one side has a lawyer or university, or there is no diversity office, the outcome will always be unjust.

### FAP trainings pose barriers to inclusion for Black trainees

Although not nearly as well-known as ACT, the FAP community is growing, with members spread around the world. At face value, it appears to be a very diverse group of empathic clinicians and researchers working to restore to CBT the heart that it seemed to have lost along the way. In fact, one of the two founders of the modality is an Asian American woman, with a fierce commitment to making the gospel of FAP available worldwide through a series of regular gatherings for the community through the MeetUp platform called Awareness, Courage, and Love (ACL). Awareness, courage, and love (also termed “strong caring” or “responsiveness”) are said to embody the key principles of FAP.

Many leaders in the FAP community hail from various Latin American countries, creating the appearance of diversity. But a closer look reveals that nearly all of these leaders are considered White in their countries of origin. It is notable that out of more than 100 certified FAP trainers globally, at the time of the writing of this paper there were no Black trainers. There were, however, a handful of Black psychologists who are nearly certified as FAP trainers. One of these “nearly certified” trainers was concerned about feedback from young Black therapists who were so disturbed by Level 1 FAP training, that they broke off or disengaged from the training process. Following interviews with those involved, it was found that the sentiments communicated by some trainees were very similar, despite absence of contact with one another. The issue had to do with the disconnect between the emotional vulnerability that the participants (Black and White) were being asked to display towards each other in the training and the violation of that vulnerability by the White participants from the onset, due to a lifetime of Western conditioning, which often denies the lived Black experience (DiAngelo, 2018; Dupree and Kraus, 2022). Some of the Black therapists who disengaged from the training expressed both reluctance to express emotional vulnerability, as well as negative effects of over-expression of emotional vulnerability in the training. Across these instances, the emotional expression of the White therapists overshadowed the experiences of the Black therapists and furthered their disengagement in the process.

It is important to appreciate that it is not the responsibility of a Black person engaged in training to educate their White classmates to prevent harm or cope with having their experiences invalidated during a training, especially given that those classmates are already therapists. But this issue recurs because many White people have been living a life which requires ignorance of racism to justify an egalitarian self-concept of non-complicity in an unfair system (Azevedo et al., 2013; Bergkamp et al., 2022; Smith et al., 2022). As such, White trainees tended to reflexively deny or minimize painful experiences of racism shared by fellow trainees of color. Correspondingly, many Black trainees felt it would be unwise to remove their armor to allow themselves to be emotionally injured in the service of experiential learning.

If the FAP leadership had ascribed to an anti-racist process, they would share power and consult Black members with more insight and empathy, and learn about the psychological dangers involved in training for certification, which is an order of magnitude more emotionally draining for Black trainees than White ones (Bergkamp et al., 2022). Issues of power and privilege have been explicated in FAP scholarship previously. Terry et al. (2010) underscore ethical mandates by explaining, “that as therapists we should become aware of power and privilege in the therapeutic context, because without intention or awareness we may be engaging in behaviors that promote inequality and injustice at the expense of our clients,” and in this case, trainees as well (p. 98). Problem-solving around this issue could look like anti-racism training instituted as a prerequisite to FAP training, which would have promoted safety and allowed Black and White participants to enter on more equal emotional footing. Although White learners will be more likely to need anti-racism training, research indicates that Black people benefit as well (Williams et al., 2020a), so this would be good for everyone (Williams et al., 2021b).

To bring these concerns to the FAP leadership, in January of 2022, one of the few Black almost-FAP trainers called a meeting of FAP leaders and several Black therapists who had completed Level 1 training. All of the Black almost-FAP trainers were present as well as two White certified FAP trainers who had helped organize the meeting. No other FAP trainers decided to attend. After describing the issue and coming up with some suggestions – among these to institute a Black-only FAP training session, the Black FAP almost-trainers asked to which Black FAP-certified trainer they could submit their suggestions, at which point it was made clear that there were no Black FAP trainers, and therefore, no one in leadership to receive or implement the suggestions. The lack of inclusiveness which became salient to both White and Black trainers at that moment left all participants discomforted and bereft of immediate solutions.

### FAP certification double standards

Interestingly, both of the well-qualified Black almost-trainers were psychologists who had completed far above and beyond all requirements, *except* the submission of a practice sample – a live recording of them conducting FAP therapy with a client. Neither psychologist felt their clients of color would be comfortable being recorded and allowing White people to scrutinize their private sessions; one felt it would be an abuse of power to ask this of their clients, who are keenly aware of the power and privilege gap between minoritized populations. As noted by Terry et al. (2010), “The therapeutic encounter consists of social behavior and its context is vulnerable to the same cultural and societal practices that empower and privilege members of certain social groups while disempowering others” (p. 110). This is why both psychologists were doubtful that White people could assess their work within its proper cultural context. Of note, other certifying bodies, such as the American Board of Professional Psychology, provide several options for providing practice samples (e.g., recordings of supervision activities) but this had not been accommodated in the FAP system. That being said, once FAP leaders became aware that their certification rules created systemic barriers to Black people being recognized as trainers, they took steps to provide alternatives to this requirement and shortly thereafter certified their first Black FAP trainer.

Completing all the requirements to become a FAP trainer is extensive and includes publishing papers and organizing/co-teaching FAP workshops. Part of the training requires the applicant to receive FAP therapy or coaching from another FAP therapist. However, the experience of one of the Black almost-trainers (Box 2) demonstrates two issues which are used to alienate and exclude Black people from positions of power in organizations; lack of empathy and policy weaponization (inequitable application or enforcement of rules based on race; Okun et al., 2019). The lack of care and empathy demonstrated in this example (Box 2) is why many Black trainees have felt reluctant or unable to engage in vulnerable FAP training with White clinicians.

BOX 2 **Perspective: an Incident of racism in a Black-White FAP trainer dyad.**

Following a one-year engagement in FAP coaching from a noted FAP trainer and author who was a White man, a prospective Black (female) FAP-trainer was met with a stunning assertion at the conclusion of her training. One of the issues she worked on with him was her fears of being unlikable, which was a by-product of her experiences as a Black woman in America. When the training was over, she took a courageous risk and told the trainer that she would like to remain friends. His response to her was both anti-reinforcing and the antithesis of their work together. He said, “No, you are too much work.” This hurtful response undermined the work she had done in trying to feel more confident and acceptable to her professional community and ultimately made it even more challenging for her to connect with the next FAP coach she had to work with, who was also White.

From an organizational perspective however, what is particularly notable is the unjust application of (unwritten) rules. When the FAP almost-trainer reported having completed the training requirements understood to be required for certification to the FAP leadership (Box 2), she was told that her coaching would not count unless she worked with a *female* trainer as well for at least 10 sessions at her own additional expense. A male colleague (White) also working toward certification, was required to do only 6 coaching sessions with only one individual in FAP leadership, provided for free. As above, this *weaponization of policy* (race-based arbitrary application of unwritten rules) is a critical reason why Black people have a harder time attaining positions of power in organizations (Okun et al., 2019; Chen et al., 2021).

Anti-racism approaches require centering the voices, opinions, and perspectives of people of color (Williams et al., 2022). Rather than power hoarding, an equitable system would invite the perspectives and presence of marginalized people and make changes to policies and procedures based on their input. On the other hand, White supremacist structures are anti-empathetic, rigid, individualistic, and do not make space for those with different lived experiences (Okun et al., 2019). Sometimes organizations may make positive changes to appear more equitable, but if White people are making the final decisions, this is still White supremacy. Psychologists should know that strong anti-racist measures are required to steer against the structural racism norms that are historic, deeply rooted, and often invisible to White people (Sue, 2017). As such the FAP certification process has been neither equitable nor anti-racist (Okun et al., 2019; Chen et al., 2021; Dupree and Kraus, 2022). The biased outcome – lack of Black certified trainers – is itself evidence of a biased process.

## ACBS shows White bias by excluding Black people from power

ACBS was developed as primarily a professional home for ACT researchers and practitioners, although related therapies like FAP have been tolerated but not similarly promoted (e.g., there is no menu tab for “FAP” on the ACBS website). Established in 2005, the ACBS website boasts 9,000 members, with slightly over half outside of the United States. There are 45 ACBS chapters covering many areas of the world including Canada, Europe, Japan, Brazil, Australia/New Zealand, Turkey, Malaysia, and more. There are also over 40 Special Interest Groups covering a wide range of areas such as children and adolescents, veteran’s affairs, ACT for Health, etc. The organizational structure is designed such that the Board maintains tight control over all committees and activities, and Board meetings are closed.

### Black people not well represented in the organization

At first glance, it appears that ACBS is a diverse organization, hailing members and organizational leaders from many countries around the globe. But on closer inspection, one notes the overrepresentation of White members, despite the international celebration, with some Asian inclusion, and little to no representation of Black people, Indigenous people, or non-White Hispanics. There are 9 members of the Board of ACBS for 2021–2022; 8 are White, 1 is Asian, and none are Black, with roughly the same pattern repeated from the inception of the organization in 2005. Every year there are 8 White people and one POC, where the POC is typically Asian and often a non-voting student member. There are no ACBS board members of color who are not Asian. There has never been a non-White president. There is no true democratic process in the election of Board members as an election committee chooses the two candidates that are able to have a run-off for each vacancy. Although the membership votes on the two choices selected by the committee, the rationale behind the choice of candidates is not made public. For the most recent voting cycle (2022), all candidates are White. It seems clear that Black people have been excluded from ACBS leadership, and Hispanic people have been critically underrepresented as well, and the organization is structured such that the membership has little power to rectify the problem.

The first associate editor of color at *Journal of Contextual Behavioral Science* (JCBS; also not Black, rather East Asian), was an ethnically Japanese person; after a long pause the next was a Southeast Asian and former student Board member only appointed recently. The first Black plenary speaker was Janet Helms who in 2019, spoke about Whiteness – much needed for the largely White audience but clearly not intended for attendees of color. Even so, one Black psychologist who was present was brought to tears, overwhelmed by finally seeing a Black psychologist honored on stage.

JCBS, the scholarly outlet for ACBS and related work, is a top-ranked journal, with an impact factor of 3.1. The journal has one editor-in-chief, 14 associate editors, 66 editorial board members, 3 student editors, and a professional officer. A review of the website reveals only a single Black person, who is on the editorial board. This means that 1.2% of the JCBS editorial board is Black, despite Black people being 12% of the US population. This is a 10x underrepresentation. More disturbingly, it is unclear the extent of involvement in ACBS of the lone Black editorial board member. This board member has a single paper published in the journal from 2015 (about body image in White women) and has not been in recent ACBS conference materials as a presenter. This could appear as tokenizing, which occurs when someone is included for the appearance of inclusion and not actually recognized for their contributions or a member of the group in a meaningful way (Williams et al., 2021d). These numbers of non-White people involved in JCBS are in line with reports and observations of poor attendance of people of color at ACBS conferences.

ACBS has a diversity and inclusion committee, however the structure of this body is different from similar professional boards. All actions must be vetted by or framed as suggestions for the Board and, in line with our previous observations about how policy can be weaponized, the Board’s decision-making processes are not transparent. The diversity committee does not get a vote or seat on the Board, and they are not permitted to attend board meetings. There has been little Black representation on this committee and only in the last few years have Black voices been included (there are currently 2 Black members). The committee has observed the Whiteness of featured speakers, mistreatment of the lone Black plenary speaker, and the shunning of Black voices at ABCS. Structurally, this committee is powerless and exists only in an advisory capacity. It is not a party to the decision-making process nor to why or how decisions are made about the very topic it was called into existence to remedy. It cannot remedy these problems and its voice is generally ignored by the Board. The lack of transparency in Board decisions, lack of power sharing with committees and members, and rigidity is consistent with White supremacist power hoarding in organizational structures (Okun et al., 2019).

### Lack of support noted by members

In December of 2015, the ACBS published the results of a Diversity Survey Report on its website. No similar survey has been done since the writing of this paper, however the results from 2015 are informative. This anonymous survey went out to the entire 7,200 members on ACBS as well as 1,700 members of ACT and 680 members of RFT listservs (Afari et al., 2015). About 10% (709) members responded, and of these, 541 had complete responses to every question. A total of 537 responders indicated their race/ethnicity with about 80% (428) identifying as White. The results do not indicate how many of the remaining 20% indicated “Black,” as the POC were lumped together into a single monolithic entity.

Although only 10% of the ACBS membership responded, the demographics are similar to what was known of the ACBS membership at the time, with a greater female to male ratio (approx. 60/40) predominantly White, US based (50%) and English speaking, with more than 50% of respondents working in clinical settings.

The report added a caveat in regard to the applicability of the findings to “minority groups” with the statement, “the majority of the respondents who gave us input about inclusivity are not necessarily diverse.” So, it is through this lens that the results should be interpreted. Nonetheless, the results of the survey do have something to say about the perception of its members about how the ACBS community is treating its non-White members. These can be seen in responses to the following questions.

ACBS asked if its community provides a supportive environment for racial and ethnic minorities. Although 70% of respondents agreed that the community was supportive of them personally, less than 40% “agreed or somewhat agreed that the community was supportive of non-English speakers, members with disabilities, nurses, physicians, those working in non-MH settings, and ethnic and racial minorities.” We find this to be a staggeringly low number. This indicates that although members (mostly White) felt supported, they were able to observe how the most vulnerable members were not. The spread between 70% and “less than” 40% is damning, especially considering that research shows that people in positions of privilege are less likely to notice the suffering of those less fortunate (Stellar et al., 2012). In a follow-up question, respondents specifically indicated that cultural diversity needed to be increased and those groups who are poorly supported and the areas of diversity which needed more work are *Cultural, Professional and Socioeconomic diversity.*

We would like to focus on the way in which the report characterized the results of the 73 comments focusing on how ACBS is not supportive of its members, who described how their experiences were demotivating and unhelpful. The report notes, “Prominent unsupportive experiences included predominating male gender, non-transparent organization, lack of cultural/developmental adaptation, predominance of English, inaccessible events, and perceived micro-aggression.” No other category was prefaced by “perceived” therefore this can be read as an indication that the writers did not believe that the microaggressions were relevant or real (or were required to use this wording to placate White leadership). Here, we would like to make the implicit racism in this phrase explicit. Notably, this report is freely available online, and Black Americans who may be interested in learning more about diversity issues as they relate to ACBS, perhaps for the purpose of joining the organization, may read this page, understand this wording to be a coded threat, and reconsider their involvement.

Finally, when asked how ACBS could improve support for diversity, 81 comments made suggestions which included: Encouraging more participation for new and diverse members, improving supervision, mentorship, and consultation for members supporting local events, and affordability as well as promoting professional diversity. After 8 years, there is no visible follow-up on how any of these suggestions may have been implemented, despite the existence of a diversity committee. The ACBS leadership understood the gravity of this feedback, but, to-date, there is no public sign of committed action on this front, and no further studies. The afore-described White supermajority Board is the body with the power to order a follow-up or make the changes advanced in the survey.

Recently, ACBS leadership produced a report of the ACBS Task Force on the strategies and tactics of CBS research, and made 33 recommendations (Hayes et al., 2021). There was very little mention of diversity issues, although Recommendation 24 stated: “CBS research needs to address diversity issues (gender; language; race, ethnicity; sexual orientation and identity, etc.) in treatment and process of change research,” with a warning about bias and noting that diversity variables need to be “thoroughly considered” (p. 180). But these recommendations rang hollow in light of the orphaned 2015 survey, a historically super-majority White board, and the absence of any acknowledgement of the human suffering caused by racism or other forms of oppression and marginalization, even within ACBS.

In our investigation, we did learn that in the aftermath of George Floyd’s extra-judicial murder and the subsequent global racial reckoning, there were shifts in the climate at ACBS that made more space for Black inclusion due to what one Black member described as “White guilt.” For example, there is a new set of clinicians of color brought in by members of the diversity committee through MEND, a group of trauma experts focused on healing communities of color^1^ using ACT. They had better experiences because the ACBS diversity committee and the related special interest group found ways to make them feel welcome, such as having a social event for people of color at a recent conference. There has been some thought and action around these issues, although members feel there is still a very long way to go in making ACBS truly inclusive.

### Is ACT relevant across races?

In a misguided attempt to showcase the diversity of ACT research, Steve Hayes (2015) posted an article on the ACBS website (listed third on Google with search terms “ACT therapy Black”) with the headline “*Does ACT work for minorities or the poor?*” The webpage and even the title is offensive, and full of implicit bias. The wording implies that racial “minorities” and the “the poor” are comparable groups that can be lumped together as if they are the same. Most Black people in the US are not poor and would be offended to be stereotyped in this way. What kind of statement would it make to have a page titled “*Does ACT work in White People or the entitled*?” or “*Does ACT work for Asian People and math geeks?*” Here we would like to make the implicit, explicit, and point out pathological stereotypes that stigmatize non-White groups. Further, the inclusion on the webpage of a study carried in South Africa on the *majority* population shows a confusion about the difference between race, ethnicity and culture. Efficacy in a majority Black, culturally and ethnically South-African population does not automatically mean that it will have efficacy in an American Black population – any more than in an ethnically Han Chinese or Indian Sri-Lankan population. Black people who are trying to learn more about ACBS will read this page and discern that this is not a safe professional home for them.

To better understand the human toll that results from racial bias, it is important to share and analyze some recent experiences that Black people have encountered within the organization. ACBS missed an important opportunity to provide allyship and support in a report of misconduct involving ACBS leaders (Box 3). White researchers published data collected by a Black researcher without the person’s consent or any acknowledgement in a contested but now published work appearing in JCBS.

This paper was the result of a collaboration between several senior primary investigators and their graduate students on a project to develop a new scale for which they agreed to share authorship. The project was set up by the investigators so that the data collected at the Black researcher’s institution would flow straight into the data collection survey system at the university of a White lead researcher.

BOX 3 **Perspective: an incident of racism against a Black scholar and ACBS member.**

In this incident, a Black researcher was reassured that after the data was collected they could be included as an author. A lead author had said by email, “There’s room for many authors as we want to be collaborative.” The Black scholar however, was shocked to later see the finished paper “in press” at JCBS. Despite intensive involvement and collecting a third of the data for the paper (over 400 subjects), the Black investigator had been completely omitted. White PIs were listed as the first several authors, followed by a number of their graduate students and junior colleagues. (To justify the exclusion of the Black scholar as an author, one of the scholar’s former graduate students of color was included as a ninth author, which is neither customary nor appropriate.)

When queried, the lead White investigator claimed in an email that it never occurred to him that the Black researcher might possess the expertise needed to meaningfully contribute to the paper – despite that the scholar had over 100 academic publications, including a dozen on psychometrics. The assertion that the scholar was underqualified is a common racial trope used to excuse exclusionary racist behavior (McDonald, 2021). What is worse is not only was the scholar excluded, but they were also manipulated into doing free work, which is another problematic racial dynamic whereby Black people are expected to work without fair compensation because their labor carries less value than labor by White people (Pettit and Ewert, 2009). The Black scholar was understandably distressed by this situation and felt exploited and deceived. Holding the position of power, the White lead authors refused to meet, compromise, or engage in mediation with the Black scholar for co-authorship, even though adding the author would not have harmed the lead authors in any way.

This authorship dispossession (Box 3) represents another example of how systemically applied weaponized policies can favor White individuals in power at the expense of a minoritized person (Gaertner and Dovidio, 2008; Okun et al., 2019; McFarling, 2021). Following a complaint to the journal, the contested article was put on hold, but the researcher was told it had to be resolved through university channels, with no help from ACBS or their ethics committee and no appreciation for the power dynamics that make it unlikely for Black persons to prevail in such disputes (Williams, 2019; Dupree and Kraus, 2022). The Black scholar had no university resources to resolve the dispute since the data was collected at their former university and they had been hired, but not yet started, at a new university. Absurdly, due to these demands, the scholar was forced to file a complaint with *the perpetrators*’ university to stall the process. In this example of policy weaponization, because they had no rules for such a situation, JCBS created *ad hoc* arbitrary rules which required that the researchers appeal to their universities. *However, it is impossible to appeal to a resource that you do not possess*. The editors used their own indifference and the ambiguity of the situation to allow a discriminatory outcome. This is aversive behavior weaponized into a policy because it allows a racist outcome on the institutional level while maintaining plausible deniability for the organization (McGillicuddy-De Lisi et al., 2006; Okun et al., 2019; Faber et al., 2023b) and uses arm’s length injustice to withhold organizational resources that could have made a difference (Okun et al., 2019; Chen et al., 2021). The incident was never reviewed or judged by an ethics or diversity committee or other impartial body. In any dispute, the institution having an interest in the outcome cannot also be the judge. As such, in a Kafkaeske process, JCBS ultimately published the paper based on the judgment of a White complaints officer at the university *of the perpetrator* without representation or a hearing for the Black researcher. As such, the data was published against their will, without acknowledgement or apology, and the journal washed their hands of the whole incident. A White lead author went on to use the data in his thesis, also without permission.

This type of issue is not an isolated incident in our field. One recent article listing multiple similar incidents to the one in Box 3 explains how the failure of White scholars to acknowledge the contribution of people of color is “intellectually dishonest and echoes a history of White people in power refusing to credit Black scholars and activists for their work” (McFarling, 2021). There is a well-documented failure to credit scholars of color, and more than enough evidence to show that publication and citation practices reproduce this institutional racism (Avery et al., 2022).

Most White people in the US and other Western nations have both explicit and implicit pro-White, anti-Black biases (Faber et al., 2019; Harjunen et al., 2021; Gran-Ruaz et al., 2022). Research shows us that White people will rarely advocate for Black people in the presence of other White people, even if this means operating against their own values (Williams et al., 2021c). Research and experience also show us that because overt racism is stigmatized, it is unlikely that the aforementioned persons are aware of the role of racism in their decision making (Williams et al., 2022). Furthermore, White solidarity will require a very high burden of proof, question over and over if racist events really happened, and look for non-racial explanations for the cause of the conflict (McGillicuddy-De Lisi et al., 2006). Persons confronted about these incidents will profess that they did not intend for the outcome to appear racially discriminatory although the outcome *is* racially discriminatory. Racism, however, does not require intent to harm people of color. Experiences such as these alert Black people and their networks to the level of care and allyship they can expect from the CBS community and contribute to low numbers of Black people in ACBS.

### ACBS and the illusion of inclusion

In an attempt to address issues such as these, the ACBS diversity committee was excited to invite an accomplished Black psychology professor to join the committee in 2019. This candidate has been a part of many organizational diversity committees in other organizations and was a founder of the Diversity Advisory Council for another large professional association. She is routinely asked to serve on diversity committees for organizations, even ones where she has not been involved.

Based on all objective criteria, such a prolific and influential scholar would be a top candidate for inclusion, however, the Board of ACBS rejected the recommendations of their own diversity committee. The justification provided to the candidate was that she had not been a member of ACBS long enough or been to enough conferences to be considered for committee membership. Yet the scholar had fulfilled all of ACBS’ specific internal qualifications for expertise in this field including a standing-room only talk at ACBS own conference, served as a grant reviewer for ACBS own grants, was a peer-reviewer for JCBS (ACBS own journal), organized an ACT (ACBS own therapy) training at Yale, and was lead author on an edited volume with New Harbinger (ACBS own publisher) about mental health equity from a CBS perspective. The rejection is consistent with the aforementioned *policy weaponization*, where unclear standards disadvantage the qualifications of Black people, regardless of how accomplished they might be due to stereotypes of inadequacy or aggression. This negative experience left the rejected Black scholar feeling further alienated from the organization, so she stopped attending conferences and eventually dropped her membership. This helped to maintain the White power imbalance by preventing a strong person of color from having even a small leadership role in the organization as it threatened the status quo.

This experience also underscores how, as aforementioned, the ACSB diversity committee suffers from a lack of power in the organization, exemplified through its inability to even choose its own members regardless of their qualifications. MIT scholars note that DEI committees “must be sufficiently empowered to implement their initiatives meaningfully and successfully” (RISE MIT, 2020). Disempowered committees cannot bring about change and are commonly used by organizations as window dressing to provide the illusion of inclusion (Okun et al., 2019).

## Discussion

Black Americans have highlighted the importance of anti-racism education and community driven practices to address the mental health needs in the Black community. The current authors contribute to these collective voices and offer to the literature our reaffirmation of the long-held position that organizations *must* confront racism and address the contemporary and historical racial paradigms within that affect Black people. Black communities emphasize that critical self-reflection at the individual level and racial equity analysis at the level of the organization is long overdue (Alang, 2019). The utility of CBS must be considered with these imperatives in mind.

However, this paper demonstrates that CBS has failed to address the needs of the Black community. This is a result of individual biases that have been enacted through the research conducted and the policies and procedures of the organization dedicated to its advancement. As key examples, the most critical mental health needs of Black people have been misunderstood and unaddressed in ACT scholarship, and Black trainees have experienced barriers to learning FAP due to lack of equity in the training process. Further, ACBS as an organization has failed to be inclusive of Black people, and it has failed to act on known problems underscored in its own investigation. Racially biased policy structures are left to exist and continue to cause racially disproportionate harm.

Psychology as a discipline is overwhelmingly White and this is not an accident (Stewart et al., 2017; Faber et al., 2023b). This disparity was pointed out in Roberts et al.’s (2020) influential paper which laid out the evidence that the topics studied, the editorial decisions, the participants in research, the influencers, professors and decision makers are overwhelmingly White. This situation influences every level of psychological science, however most profoundly, results in a state of self-deception, an inability for the beneficiaries of this White-biased system to impartially see and quantify the problems, and rose-colored glasses about the effects and outcomes of this Whiteness. More succinctly put, “White people benefit from obscuring the existence of racial inequality from which they benefit” (Bergkamp et al., 2022; Dupree and Kraus, 2022, p. 271). Pointing out facts like these generates defensiveness, which functions to deflect attention from the real issues (Howell et al., 2017; DiAngelo, 2018).

One of the main tasks for anti-racist advocates is to drag hidden and covert racism into the light, not so that people can be shamed but so that problems can be seen and resolved. This is an essential pathway to healing and reconciliation with non-White communities. That which cannot be seen cannot be treated. This mindset is fundamentally at odds with White supremist structures that benefit from racism, particularly when it remains unacknowledged, stigmatized, or covert (Okun et al., 2019). As researchers and practitioners, we understand how and why it is not in the best interest of therapists or clients to allow these ingrained cultural impulses to go unchecked. Without transparency, Black practitioners and researchers will continue to be at odds with ACBS.

Further, misconduct and reports of discrimination should be monitored and mediated by the ACBS community. It is critical that some level of protection and oversight is put in place to remedy the ongoing enactment of racism in its professional circles. As psychology researchers, senior voices in the field should be well-aware that structural racism remains embedded in every structure from client care to membership to the publication process, to review and be ready to confront racism as it arises (Williams, 2019; Dupree and Kraus, 2022). There is no naturally occurring racial progress. Gains are brought about only by struggle (Dupree and Kraus, 2022).

It can be expected that some who read this will rightly assert that it is easy to point out problems but hard to come up with solutions. We do have a solution, and it can be summarized succinctly as *power-sharing*. In Table 2, we provide some practical examples of steps that can be taken to make CBS spaces more inclusive and equitable. If ACBS is interested in systemic organizational changes, they can take note of these anti-racist steps being taken by many similar professional organizations, in particular the apology of the APA to People of Color for their failure to challenge racism within the organization (APA Council of Representatives, 2021). Notably, other organizations with similar problems might also implement these solutions where applicable.

**Table 2:** Practical steps for anti-racist organizational transformation.

Governance	Revise ACBS organizational bylaws to require 50% of all board and committee seats occupied by people from marginalized groups (including Black people).
	To increase transparency, ACBS can post board minutes on the organizational website (redacted where needed for confidentiality).
	Increase the ethnic and racial diversity of the journal’s editorial board composition.
	Implement anti-racist practices in JCBS editorial processes, including appointing designated associate editors with expertise in diversity issues (as was done by the Association for Behavioral and Cognitive Therapies; Sanders et al., 2022).
	Empower the ACBS diversity committee to improve their ability to affect change in the organization (e.g., they appoint their own members, are provided with a budget and funding for projects, have at least one DEI-informed board member who actively participates in the committee, etc.)
	Have open and free elections (i.e., rank ordered vote for Board seats and officers), based on members getting signatures of other members in support of their candidacy, for example, rather than having candidates hand-picked by the current super majority-White Board.
	Hold Annual General Meetings (AGMs) with ACBS members, to allow the Board to report back to the membership about their progress towards the mission and strategic priorities of the organization, including equity goals, and to encourage dialogue and discussion within the ACBS community on key issues affecting members.
	Anti-racism and organizational culture change is everyone’s responsibility. Equity and anti-racism goals should be woven into ACBS’s foundational documents, policies, and practices (i.e., how we put our values into practice as a goal in the strategic plan, reporting on progress in annual reports, developing operational policies that explicitly reference equity and inclusion as core to the functioning of the organization, etc.) rather than sitting as a stand-alone initiative.
Metrics	Collect racial, ethnic, and other identity data to quantify disparities at ACBS (membership, grant recipients, conference attendees, etc.) and be able to create a baseline to track progress moving forward (ABCT Board of Directors, 2021).
	Collect racial, ethnic, and other equity seeking data on JCBS editors, reviewers, and authors to ensure equity in publishing and to track progress moving forward (Else and Perkel, 2022).
	Re-administer the 2015 survey, use it as a springboard for updating Specific, Measurable, Achievable, Realistic, and Timed (SMART) goals, led by the diversity committee (Bjerke and Renger, 2017).
Research	Organize a special issue of JCBS focused on research about non-White groups (as done by APA and APS; Wolitzky-Taylor et al., 2017; King, 2021).
	Provide annual grants and awards for CBS research focused on people of color and researchers of color (as done by the American Board of Professional Psychology).
	Require all submission to the organization’s journal to include an author positionality statement and sample race and ethnicity data (with an explanation for non-diverse samples).
Teaching	Organize listening forums where leadership will hear the concerns of Black members to direct change [as was done by the National Institutes for Mental Health (NIMH; Gordon, 2022)].
	Implement annual anti-racism training for all Board members and those in leadership positions.
	Organize a conference focused on issues impacting non-White groups (i.e., Kouame et al., 2021).
Recon-ciliation and mitigation	Publish a Board statement apologizing for perpetuating racism in psychology, as done by the APA Council of Representatives (2021), and outline the initiatives being implemented to change the situation.
	Task the diversity committee or a DEI consultant with reviewing all rules, policies, practices and procedures to identify and excise institutional racism and propose updates to ensure the organization has the internal infrastructure needed to become more equitable.
	Reach out to Black people who have left the organization and find out why they left and what it would take to meet their needs and be able to return to the ACBS community.
	Create a confidential process to investigate complaints of discrimination whereby members can choose a liaison or investigator from their own identity group (Charkoudian and Wayne, 2009).
	Provide free ACBS membership to Black and other marginalized people until the organization achieves representative levels of diversity in its membership.
	Add current, useful, and non-demeaning diversity content to the ACBS website with help from the diversity committee (as was done by the International OCD Foundation; Gimber, 2020).

## Conclusion

To date, there are no signs that the warning bells of the ACBS 2015 diversity survey have been followed by relevant actions or even a new survey. Further, there does not seem to be any accountability structure toward POC in regard to culturally-informed research, anti-racist practice, diversity grants, publishing, certification, or ethical behavior in psychology as it pertains to executive leadership, decision-making, and structural organization. Anti-racism approaches require an honest examination of the problems, despite discomfort and without experiential avoidance.

Existing structures will resist change, and as such implementing equity will take courage and persistence. This may be uncomfortable, but within this solution is also where ACT and FAP principles can be helpful. Those who care to do better can *accept* they have been participating in a racist system, *accept* the unpleasant feelings that accompany that level of honesty, *commit* to becoming anti-racist allies, and take valued actions to create equity. They can become more *aware* of their impact on Black people, have *courage* to make a change, and show *love* by being better human beings through tangible acts of care (Tsai et al., 2009; Williams et al., 2022).

## Author contributions

SF and MW contributed primarily to the conception and overall writing. IM was involved in refining and editing the text as well as adding commentary on critical events narrated in the text. JT drafted the sections on FAP and ACT as well as checking the references, and CF contributed to the early drafts to the first half of the paper on the needs of the Black experience. All authors contributed to the article and approved the submitted version.

## Footnotes

^1^ www.mendminds.org
